# New advances in efficacy prediction of extracorporeal shock wave lithotripsy in pediatrics: a narrative review

**DOI:** 10.3389/fped.2025.1681384

**Published:** 2026-01-21

**Authors:** Mingyi Zang, Yang Dong, Xitao Wang, Conghui Han, Jianye Jia

**Affiliations:** 1 Department of Urology, Xuzhou Clinical School of Xuzhou Medical University, Xuzhou Central Hospital, Xuzhou, Jiangsu, China; 2 Department of Urology, Peking University International Hospital, Beijing, China

**Keywords:** artificial intelligence, efficacy prediction, extracorporeal shock wave lithotripsy, pediatric urolithiasis, stone density

## Abstract

Extracorporeal Shock Wave Lithotripsy (ESWL) has been a cornerstone in treating pediatric urinary stones for nearly four decades, but requires tailored approaches due to anatomical and physiological differences from adults. This review synthesizes current evidence on ESWL efficacy predictors in children, integrating multicenter data and emerging technologies. Key traditional predictors include favorable stone characteristics [density ≤600 Hounsfield units [HU], size ≤15 mm, skin-to-stone distance [SSD] ≤6.6 cm, upper/middle calyx or ureteral location] and patient factors (age ≤3 years, male sex); conversely, urinary tract infections (UTIs), BMI >22, and multiple stones correlate with poorer outcomes. Innovations like dual-energy CT (DECT), AI-based models, shear wave elastography (SWE), and bioelectric impedance analysis (BIA) offer promising non-invasive preoperative assessment. We highlight the need for standardized multifactorial predictive models to optimize pediatric ESWL outcomes. Future directions emphasize AI, big data, and multidisciplinary collaboration to enhance personalized treatment and reduce complications. This analysis provides clinicians with evidence-based tools to refine pediatric ESWL protocols.

## 1 Introduction

Extracorporeal Shock Wave Lithotripsy (ESWL) has been employed for treating pediatric urinary stones for nearly four decades (1). While ESWL was quickly adopted as a non-invasive modality in adults, its application in children has been approached with greater caution due to significant anatomical and physiological differences in the pediatric urinary system (2, 3). Consequently, the direct application of adult treatment protocols to children may be inappropriate (4). Developing pediatric-specific efficacy prediction models that integrate adult-derived research with child-friendly characteristics is therefore essential.

Research on predictors of pediatric ESWL efficacy remains incomplete, with key indicators insufficiently evaluated. To maximize therapeutic outcomes while minimizing complications, comprehensive patient assessment and thorough analysis of major influencing factors are critical. This review systematically synthesizes efficacy predictors derived from adult studies, integrates evidence-based medicine and clinical experience, and explores their applicability in pediatric populations.

## 2 Study design

This is a narrative review that systematically synthesizes evidence on predictive factors for ESWL efficacy in pediatric urolithiasis. We integrated data from multicenter studies, observational studies, and technical innovations, focusing on both traditional predictors and emerging technologies.

## 3 Search strategy

We conducted a systematic literature search in PubMed and MEDLINE databases for articles published between February 2005 and February 2025. The search strategy used a combination of MeSH terms and free-text keywords: (“extracorporeal shock wave lithotripsy” OR “ESWL”) AND (“stone-free rate” OR “Hounsfield unit” OR “skin-to-stone distance” OR “body mass index”) AND (“pediatrics” OR “children”) AND (“predictors” OR “prognostic factors”).

Inclusion criteria: (1) peer-reviewed articles; (2) human subjects aged ≤18 years; (3) English language; (4) focus on predictors of ESWL success in pediatric urolithiasis.

Exclusion criteria: (1) case reports, editorials, or review articles (to avoid duplicate evidence); (2) studies involving adult populations only; (3) non-urologic applications of ESWL; (4) insufficient data on predictive factors.

Additionally, the reference lists of retrieved articles were manually screened to identify any further potentially eligible studies (Table 1).

**Table 1:** New advances in pediatric extracorporeal shock wave lithotripsy efficacy prediction studies.

Section	Factor	Author
Traditional predictors and assessments of ESWL efficacy in adults	Triple D score	Tran et al.
	Quadruple D score	Ichiyanagi et al.
	Kanao's nomogram	Kanao et al.
Predictors and assessment of pediatric ESWL efficacy	Sendogan's study	Sendogan et al.
	Bulut's study	Bulut et al.
	El-Assmy's study	El-Assmy et al.
	McAdams's study	McAdams et al.
New insights into predictors and Assessment of Pediatric ESWL Efficacy	Age	Jia et al.
		Jayasimha et al.
		Wagenius et al.
		Onal et al.
	Gender	Soleimani et al.
		Dogan et al.
	Medical history and laboratory tests	Demirtas et al.
		Wang et al.
		Caltik et al.
		Ripa et al.
		Manzoor et al.
		Li et al.
		Moyer et al.
		Lu et al.
		Schuil et al.
		Zheng et al.
	Stone size	Abid et al.
	Stone location	Kızılay et al.
		Jia et al.
	Stone density	Tu et al.
	SSD and BMI	Sendogan et al.
		El-Assmy et al.
		Kizilay et al.
	Number of stones	Jayasimha et al.
		Onal et al.
		Dogan et al.
		Jia et al.
New predictive methods	Imaging histology and computer science	Yang et al.
		Akkas et al.
	Shear wave elastography	Samir et al.
		Demir et al.
		Taljanovic et al.
		Urban et al.
	Bioelectric impedance analysis	Keser et al.
		Ferguson et al.

BMI, body mass index; SSD, skin-to-stone distance.

Due to the heterogeneity of study designs (e.g., retrospective observational studies, multicenter cohorts) included in this narrative review, we did not perform formal methodological quality assessment (e.g., Newcastle-Ottawa Scale for observational studies). Instead, we prioritized studies with large sample sizes (>50 patients), clear outcome definitions (stone-free rate ≥3 months post-treatment), and transparent reporting of predictive factors to enhance the reliability of our conclusions.

## 4 Traditional predictors and assessments of ESWL efficacy in adults

A systematic PubMed search up to February 2025 identified three representative scoring systems for predicting preoperative ESWL efficacy in adults: the Triple D score [area under the curve (AUC) = 0.834], the Quadruple D score (AUC = 0.651), and the preoperative nomogram proposed by Kanao et al. (5–7). These models predominantly utilize anatomical and imaging parameters, including maximum stone diameter/volume, anatomical location, stone number, computed tomography (CT) attenuation values, and skin-to-stone distance (SSD). These parameters assist clinicians in assessing the complexity of stone fragmentation and establishing a quantitative framework for guiding therapeutic strategies. Although these adult-derived prediction models offer valuable reference points for pediatric research, children's anatomical and physiological profiles demand further refinement and adaptation of these systems to suit pediatric treatment protocols.

## 5 Predictors and assessment of pediatric ESWL efficacy

A comprehensive search of the PubMed and MEDLINE databases (up to February 2025) identified four multicenter studies (Table 2) evaluating at least three predictive factors for ESWL efficacy in 415 pediatric patients (age 1–16 years, male-to-female ratio ≈ 1.3:1, mean age 7.5–8.5 years, stone diameter 3.8–20 mm). Reported ESWL success rates ranged from 63% to 90.2%.

**Table 2:** Multicenter studies evaluating predictors of ESWL efficacy in pediatric patients.

Study	Characteristics	Stone density(HU) cutoff	SSD(cm) cutoff	Stone size cutoff
Sendogan et al.	158 children, renal stones	540	6.7	150 mm^3^
Bulut et al.	147 children, renal stones	550	6.5	155 mm^3^
El-Assmy et al.	57 children, renal stones	600	–	12 mm (length)
McAdams et al.	53 children, renal stones	1,000	–	–

Values represent cut-off thresholds used in predictive models.

These studies consistently employed stone density (540–1000 HU) as a key predictor of ESWL success. Other commonly assessed variables included SSD and stone size. Optimal SSD thresholds ranged from 6.5 to 6.7 cm, while stone size cutoffs were set at 150–155 mm^3^. Notably, El-Assmy et al. employed a stone length ≤12 mm to define the size threshold (8–11).

Stone density, SSD, and stone volume are significantly associated with ESWL prognosis. Comprehensive studies indicate that stone densities below 600 HU are linked to higher treatment success, while values exceeding 1,000 HU significantly increase the risk of failure. Moreover, a shorter SSD (<6.5 cm) and smaller stone size (volume < 150 mm^3^ or length < 12 mm) are correlated with better ESWL efficacy in pediatric patients. To enhance clinical decision-making, pediatric ESWL prediction models should be refined to incorporate these key parameters: stone density, SSD, and stone size.

## 6 New insights into predictors and assessment of pediatric ESWL efficacy

Stage-specific patterns, continuity, asynchrony, and individual variability characterize pediatric growth and development. Based on current evidence, key clinical predictors and assessment methods were synthesized to influence ESWL efficacy in children.

### 6.1 Age, gender

Jia et al. (12) conducted a systematic analysis of 300 children with upper urinary tract stones treated with ESWL between March 2009 and July 2010. The cohort (211 men, 89 women) ranged in age from 7 months to 10 years, with 73.33% under 3 years. Their findings indicated that younger age correlated with superior ESWL success (except cystine stones), a finding consistent with multiple studies (13–15). This trend may be attributed to shorter stone formation time, lower density, and softer texture in younger patients, except in the case of cystine stones, which respond poorly to ESWL.

Soleimani et al. (16) analyzed 144 pediatric patients (<14 years) treated with ESWL for upper urinary tract stones at their institution in 2018. The cohort comprised 41% males (59/144) and 59% females (85/144), indicating a significantly higher proportion of female patients. The overall post-ESWL stone-free rate (SFR) was 91% (131/144), with men demonstrating substantially higher success rates than women (98.3% vs. 85.9%), consistent with findings from Dogan et al. (17). Collectively, these studies indicate that male pediatric patients may experience better therapeutic outcomes with ESWL. This gender-based disparity in efficacy may be mediated by anatomical factors (such as ureteral anatomical variations) or hormonal influences affecting stone fragmentation. Future studies should adjust for stone location and number to confirm the independent effect of sex on ESWL efficacy.

### 6.2 Medical history and laboratory tests

Demirtas et al. (18) reported that 55% of pediatric patients with urinary stones in outpatient settings had concurrent urinary tract infections (UTIs), with 23% diagnosed at admission, substantially higher than rates observed in adults. However, published reports on pediatric stone disease with concurrent UTI remain limited. Based on clinical observations and relevant literature, the following aspects are noteworthy:

#### 6.2.1 Diagnostic criteria for pediatric urolithiasis with urinary tract infection (UTI)

Symptom reporting is frequently unreliable in children (particularly infants and toddlers) unless accompanied by systemic signs such as fever. Urinalysis remains central, where urine color and odor can provide early indications. However, isolated pyuria has limited diagnostic value for UTI complicated by urolithiasis. Furthermore, urine cultures may be compromised by contamination, and results frequently differ between bladder urine, renal pelvic urine, and stone cultures (19–21).

#### 6.2.2 Influence of bacteria on stone formation in children

Urease-producing bacteria are definitively linked to infection-related stones, specifically struvite (magnesium ammonium phosphate hexahydrate). The role of non-urease-producing bacteria (such as *Escherichia coli* and *Enterococcus spp*.) remains unclear. Hypothesized mechanisms, mostly derived from adult studies, include renal epithelial injury and inflammation (enhancing crystal retention), altered urinary microenvironments due to bacterial metabolism, and biofilm formation (serving as a nidus for stone matrix deposition). Notably, children with infection-associated stones frequently exhibit reduced urinary citrate levels (22–24).

#### 6.2.3 Impact of infection on perioperative complications

Empirical antimicrobial use in patients with unclear diagnoses, particularly those with indwelling ureteral stents, is frequently inappropriate. Indiscriminate antimicrobial administration under such conditions may promote the development of drug-resistant organisms. This issue is further complicated by the limited range of antimicrobial agents approved for pediatric use, making perioperative antimicrobial selection particularly challenging. To mitigate antibiotic overuse, clinicians should prioritize evidence-based decision-making, integrating comprehensive clinical histories and validated laboratory data. This approach enhances patient safety and reduces the risk of procedure-related complications (25, 26).

#### 6.2.4 Effect of infection on stone recurrence after ESWL

There are currently no established guidelines or consensus regarding postoperative antimicrobial protocols for pediatric patients with infection-related stones. Consequently, further research is imperative to determine optimal antimicrobial strategies following ESWL to ensure adequate infection control and reduce recurrence rates.

Moyer et al. (27) conducted a retrospective analysis of 111 pediatric patients (≤ 17 years) treated for urinary stones at two children's hospitals in Wisconsin between 2012 and 2017. Initial 24 h urine metabolic testing revealed abnormalities in 62.2% (69/111) of cases. The prevalence of metabolic disorders was significantly higher in men than women (81% vs. 48%). Hypercalciuria (55%) and hypocitraturia (73%) were the most common abnormalities, consistent with prior research. Our clinical experience supports the utility of 24 h urine metabolic testing in high-risk children to identify underlying etiologies, assess recurrence risk, and guide personalized interventions. However, widespread implementation is currently limited by high costs and technical complexity.

### 6.3 Stone size

For pediatric upper urinary tract stones, ESWL is typically recommended when the stone diameter is <15 mm. Study data demonstrate SFRs of 78.7% for stones <10 mm, 77.8% for 11–15 mm, and 66.6% for 15–20 mm (28), revealing a clear inverse correlation between stone size and treatment success. This progressive SFR decline (from 78.7% to 66.6%) underscores the importance of size-based patient selection.

### 6.4 Stone location

**Figure 1:**
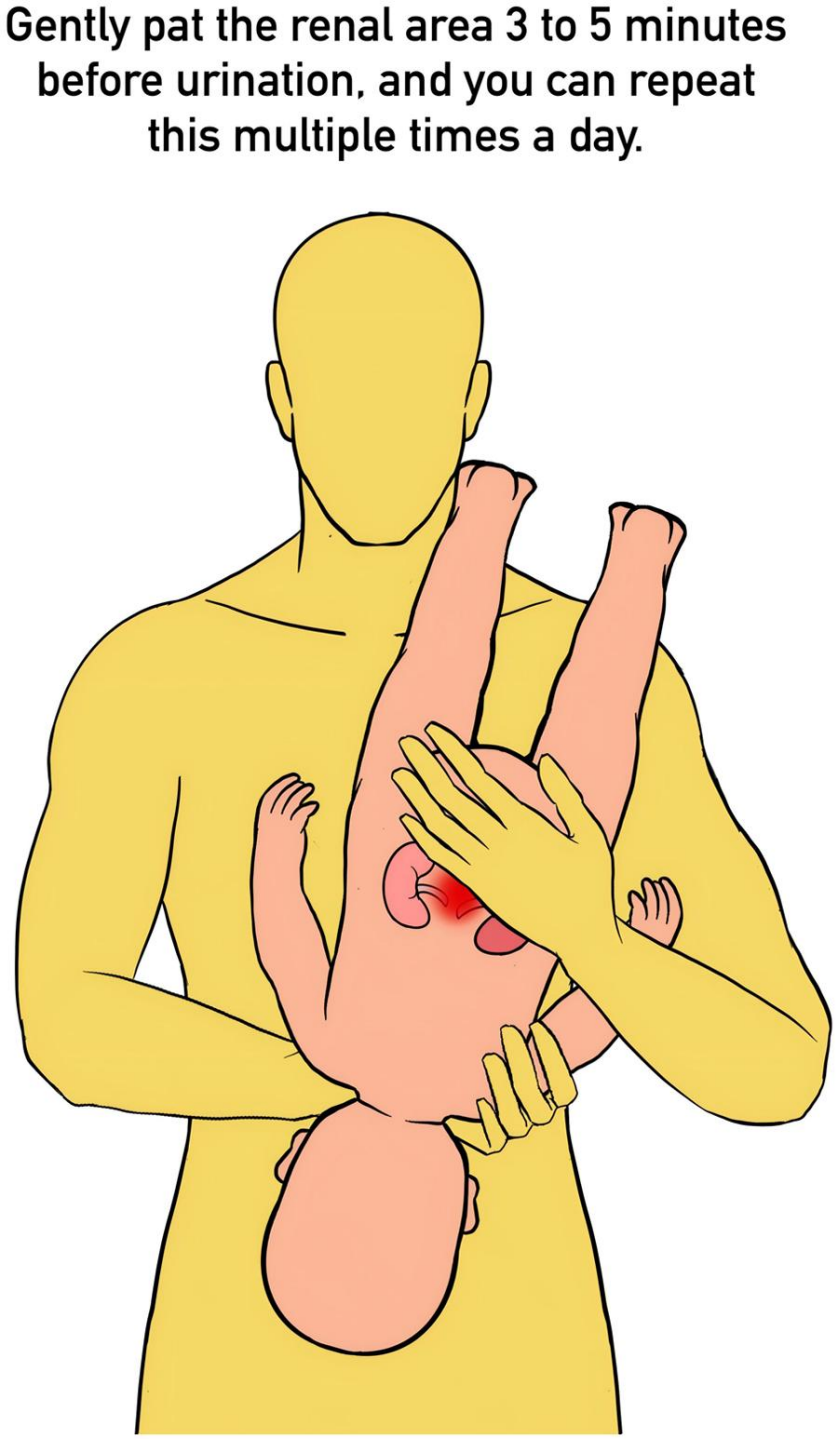
Inverted drainage positioning: Jia et al. found that even for lower pole stones, a high stone clearance rate could be achieved if correct positioning techniques were employed.

In a study by Kizilay et al. (29), preschool and school-aged children (mean age: 6.6 years) undergoing ESWL demonstrated stone clearance rates of 52.2% for lower pole calculi, with significantly higher rates for upper pole (86.6%) and middle pole (72.3%) stones. In another study by Jia et al. (12) involving 300 pediatric patients (mean age: 34.9 months), ultrasound localization was adapted based on age, body size, and stone location. Operators stabilized the lumbosacral area with arm support, ensured proper balloon inflation, optimized probe-skin contact, and adjusted treatment parameters for accurate targeting. Techniques like inverted positioning for lower pole stones helped address anatomic challenges. Clearance rates were 88.57% for the upper pole, 95.55% for the middle pole, and 91.83% for the lower pole stones, with minimal residual fragments in lower pole cases. Ureteral stones showed high clearance rates (proximal: 97.1%, mid: 100%, distal: 100%) without significant differences. For differences in results, Jia et al. demonstrated that in children under 3 years old undergoing ESWL for upper urinary tract stones, treatment efficacy was less affected by stone location compared to older children. Furthermore, they employed innovative techniques such as inverted drainage positioning (Figure 1), achieving a higher stone-free rate for lower calyceal stones. This narrowed the gap in stone-free rate between different stone locations and improved overall lithotripsy outcomes.

### 6.5 Stone density (CT value)

Computed tomographic (CT) scanning remains the standard for assessing urinary stone composition. CT attenuation values (measured in Hounsfield units, HU) reflect stone density and indirectly estimate hardness, aiding in ESWL outcome prediction. Currently, dual-energy computed tomography (DECT) offers distinct diagnostic advantages for stone analysis. The DECT captures simultaneous high- and low-energy x-ray images, generating two separate CT attenuation values. The ratio of these values enables partial differentiation of the stone's chemical composition.

A study by Tu et al. (30) demonstrated a considerable correlation between DECT parameters and pediatric ESWL outcomes. ROC curve analysis identified optimal CT thresholds of 882.5 HU (at 140 kVp) and 1,330.5 HU (at 80 kVp) for the dual-energy index (DEI), with corresponding AUC values of 0.780 and 0.766. These findings support the use of DECT parameters in predicting ESWL success, achieving a model accuracy of 95%, thereby providing substantial evidence for clinical implementation.

A study from the University of Minnesota Medical Center similarly utilized CT attenuation to predict ESWL outcomes. Patients with stone density < 1,000 HU demonstrated a 77% success rate, while those with densities > 1,000 HU had a markedly lower rate of 33%, confirming a significant correlation between preoperative CT values and ESWL efficacy in children.

Even stones with identical chemical composition may differ in internal crystalline structure and molecular alignment, leading to variations in density and hardness. These differences become more pronounced between chemically distinct stones, where crystal lattice organization and molecular binding forces result in different physical properties and varied responsiveness to shockwaves.

### 6.6 Skin-to-stone distance (SSD) and body mass index (BMI)

The SSD represents the shockwave energy transmission pathway in ESWL, with greater distances causing more energy attenuation. SSD in children varies significantly by age. In Sendogan et al.'s study (mean age: 8.5 ± 3.5 years, *n* = 158), the stone-free group demonstrated a substantially shorter SSD (6.3 ± 0.5 cm) compared to the residual stone group (7.0 ± 0.4 cm) (9). Conversely, El-Assmy et al. (mean age: 5.4 ± 3.7 years, *n* = 57) reported no significant SSD difference between success and failure groups (4.1 ± 1 cm vs. 3.9 ± 0.88 cm) (10), diverging from adult data. These findings suggest that reduced subcutaneous fat in younger children minimizes SSD variability, consequently diminishing its impact on ESWL efficacy.

While advancements in ESWL technology (such as improved targeting accuracy and energy delivery) have mitigated the impact of SSD on treatment efficacy, the rising prevalence of pediatric obesity presents a growing clinical challenge. This issue stems from the limited penetration depth of many lithotripters. When the focal zone is too shallow, excess abdominal fat and muscle tissue significantly attenuate shockwave energy before it reaches the stone. Kizilay et al. (29) demonstrated substantially higher BMI values in the ESWL failure group (24.2; range: 23.6–28.6) compared to the success group (19.4; range: 17.8–22.5). As a validated indicator of obesity, BMI serves as a crucial predictor of ESWL outcomes, particularly in contemporary pediatric populations with increasing adiposity.

### 6.7 Number of stones

Jayasimha et al.'s retrospective analysis of 76 pediatric ESWL cases in India revealed a considerably higher failure rate for multiple stones (72.73%) compared to solitary stones (27.27%) (13). In a study of 395 children, Onal et al. reported SFRs of 88% for single stones vs. 32% for multiple stones after initial treatment, with multifocal stones requiring an average of 3.12 sessions for complete clearance (15). Similarly, Dogan et al. found first-session success rates of 62.7% for solitary stones and 32.4% for multiple stones among 383 pediatric patients (17). A study by Jia et al. (*n* = 189, mean age: 22.80 ± 7.14 months) confirmed this trend, reporting a 97.88% SFR for single stones in infants/toddlers, significantly higher than for multiple stones (31).

Based on our experience, caregivers of children with multiple stones should be counseled about the likelihood of requiring repeated ESWL sessions. Repeat treatments should not be considered a failure but rather part of the therapeutic continuum. A minimum interval of 3–4 weeks between sessions is recommended. For complex cases, clear communication and realistic expectations regarding multi-session treatment plans are essential. Excessive accumulation of shockwave energy or the delivery of too many shocks increases the risk of tissue damage surrounding the stone.

Based on current literature and our clinical research, a practical tool was delivered to facilitate physicians in rapidly evaluating the prognosis of pediatric ESWL treatment. An innovative Predictive Evaluation Table (Table 3) enables physicians to estimate the likelihood of treatment success by assessing key predictive factors. This tool provides a scientific foundation for formulating individualized treatment plans.

**Table 3:** Predictive evaluation scale for pediatric ESWL efficacy.

Factor	Favorable	Unfavorable
Age	≤3 years	>3 years
Gender	Male	Female
UTI	Absent	Present
Stone diameter	≤15 mm	>15 mm
Stone location	Upper/middle calyx or ureter	Lower calyx
Stone density (HU)	≤600 HU	>600 HU
SSD	≤6.6 cm	>6.6 cm
BMI	≤22	>22
Stone number	Single	Multiple

This scoring tool has not been prospectively validated in clinical practice and should be considered hypothesis-generating.Pediatric BMI should be interpreted using age- and sex-adjusted growth charts/percentiles, while values around 22 and above may indicate obesity particularly in school-aged children. Pediatric obesity (age-adjusted)-rather than a single number-is associated with reduced ESWL success.

## 7 New predictive methods

### 7.1 Imaging histology and computer science

Artificial intelligence (AI) has recently made significant strides in medical imaging and prediction. Yang et al. (32) developed an AI system that automatically extracts and analyzes CT parameters from patients with pediatric stones to predict ESWL outcomes, replacing traditional manual measurement. This approach conserves time and resources but also demonstrates superior predictive accuracy [area under the receiver operating characteristic curve (AUROC) = 0.83]. Moreover, Akkas et al. (33) established a decision-making model for assessing the suitability of ESWL based on stone location, further enhancing treatment planning and precision.

### 7.2 Shear wave elastography (SWE)

Shear wave elastography (SWE) demonstrates a significant correlation with CT HU, making it an effective alternative for assessing stone density. SWE offers distinct advantages, including lower cost and the elimination of radiation exposure, addressing safety concerns associated with pediatric CT imaging. For predicting ESWL outcomes, an SWE threshold of ≥15.5 kPa yields a 72.2% sensitivity and 71.8% specificity, supporting its reliability as a predictive tool (34). These findings highlight SWE as a viable non-radiative alternative to CT for preoperative evaluation of stone density and treatment efficacy in pediatric lithiasis (35–37).

### 7.3 Bioelectric impedance analysis (BIA)

Bioelectric impedance analysis (BIA) is a non-invasive technique that measures body composition (fat, muscle, water content) using low-intensity electrical currents. Its simplicity, radiation-free nature, and high reproducibility make it well-suited for pediatric patients with urinary stones. Among BIA-derived parameters, body fat percentage (BFP) significantly impacts ESWL outcomes. According to predictive models, each 1% increase in BFP reduces the likelihood of treatment success by 6.7%, indicating a strong inverse correlation between adiposity and ESWL efficacy (38, 39).

## 8 Clinical summary and future directions

Key Predictors of Success: Favorable factors include smaller stone diameter (≤15 mm), ureteral and then non-lower-pole renal location, lower stone density (≤600 HU), and shorter skin-to-stone distance (≤6.6 cm).

Important Patient Factors: Younger age (≤3 years) and male sex are positive predictors, while pediatric obesity (based on age and sex-adjusted percentiles) and urinary tract infection correlate with lower success rates.

Emerging Technologies: AI, SWE, and BIA are promising for future risk stratification but require prospective validation before clinical routine use.

Future Directions: In recent years, pediatric ESWL has developed rapidly, with an increasing number of regions and hospitals establishing related programs. It is imperative to conduct more research on pediatric ESWL. Such efforts should focus on integrating advanced technologies to develop a comprehensive and accurate predictive scoring system for treatment efficacy, which must subsequently be validated across broader populations and diverse ethnic groups of children.

## 9 Limitations

Currently, there is still insufficient research on efficacy prediction of pediatric extracorporeal shock wave lithotripsy, with a lack of relevant consensus and meta-analyses, which requires the joint efforts of clinicians and researchers worldwide.

For novel technologies such as AI, SWE, and BIA, the level of evidence is still limited. Further research is required to develop these promising technologies.Additionally, practical constraints need to be acknowledged: implementation costs for specialized equipment and software are non-trivial, making them less accessible in resource-limited settings; radiation exposure (relevant for AI-integrated CT-based tools) remains a concern for pediatric patients; and clinicians may need additional training to effectively use these technologies, as they involve specific operational and interpretive skills. These factors call for targeted optimization in future research.

This scoring tool (Table 3) has not been prospectively validated in clinical practice and should be considered hypothesis-generating.
